# Assessing Gender Equality in Italian Animal Science: A Case Study on Academic Careers and Research Outcomes

**DOI:** 10.3390/ani15030390

**Published:** 2025-01-30

**Authors:** Anna Sandrucci, Lucia Bailoni, Paola Crepaldi

**Affiliations:** 1 Department of Agricultural and Environmental Sciences- Production, Landscape, Agroenergy, University of Milan, 20133 Milano, Italy; anna.sandrucci@unimi.it (A.S.); paola.crepaldi@unimi.it (P.C.); 2 Department of Comparative Biomedicine and Food Science, University of Padova, 35020 Legnaro, Italy

**Keywords:** academic careers, authorship, bibliometrics, gender gap, segregation

## Abstract

This paper focuses on gender issues within the Italian Animal Science and Production Association (ASPA), which includes both academic and non-academic researchers involved in animal sciences. Data from the association’s archives in 2023 and bibliometric data obtained from Scopus and Web of Science were used. This study highlighted how within the ASPA and in that part of the academic realm falling within the field of animal science, a noticeable gender gap still exists, albeit decreasing over time at a very slow pace. There is clear vertical segregation, with a minority of women occupying full professor positions, and horizontal segregation with a low percentage of women involved in animal science disciplines closer to STEM fields, such as “Animal breeding and genetics”. At the bibliometric level, gender differences in bibliometric indicators are observed, favouring men, although these differences are not significant when considering the academic position, scientific sector, and age. In the official journal of the ASPA (*Italian Journal of Animal Science*), women are under-represented in prestigious positions such as first, last, and corresponding authors. Scientific societies, academic institutions, and research organizations are called upon to do their part to accelerate the process of overcoming these inequalities.

## Simple Summary

Gender equality is a goal (Goal 5) of the United Nations Development Programme, yet challenges persist in academia, especially in fields like animal science. This study focuses on gender issues within the Italian Animal Science and Production Association (ASPA), which includes both academic and non-academic researchers involved in animal sciences. Using 2023 data from ASPA and bibliometric information from Scopus and Web of Science, the research highlights a slow but ongoing reduction in the gender gap. However, vertical segregation remains a major issue, with few women holding top positions like full professors. There is also horizontal segregation, as fewer women work in animal science areas closely related to Science, Technology, Engineering, and Mathematics (STEM) disciplines, such as Animal breeding and genetics. At the bibliometric level, men generally perform better in research metrics like publication and citation counts. However, these differences become less significant when considering academic position, scientific sector, and age. Women are still under-represented in prestigious authorship.

## 1. Introduction

Gender equality constitutes one of the 17 key objectives (Sustainable Development Goals, SDG) outlined in the 2030 Agenda for Sustainable Development, signed by 193 United Nations (UN) member states in 2015. As highlighted in SDG 5, “Gender equality is not only a fundamental human right but a necessary foundation for a peaceful, prosperous and sustainable world”. This commitment has to be actively pursued by recognizing and enhancing female skills while ensuring equal leadership opportunities at all levels of the decision-making process. UNESCO also recently published a document proposing global action to address the persistent gender gap in science [1].

In recent years, considerable progress has been achieved and regulatory interventions have emerged. In the United States, the National Strategy on Gender Equity and Equality [2] is the first comprehensive national strategy to advance gender equity and equality. It is built on an intersectional approach that acknowledges the overlapping forms of discrimination related to gender, race, sexual orientation, and socioeconomic status, among other factors, identifying ten interconnected priorities. The EU has implemented comprehensive strategies for gender equality, resulting in the formulation of the Gender Equality Strategy 2020–2025. The Strategy, defined as “a set of commitments and actions that aim to promote gender equality in an organization through a process of structural change”, presents policy objectives and actions to make significant progress by 2025 toward a gender-equal Europe [3]. Despite the presence of guidelines in both national and international regulatory frameworks, achieving gender equality in the workplace and professions remains a significant challenge. The Global Gender Gap Report 2024 from the World Economic Forum highlights that significant gender disparities persist [4]. Progress towards closing the gap has been extremely slow, with a slight improvement compared to 2023. At the current pace, it is estimated that it will take 134 years to achieve full gender parity globally. This report highlights significant gender disparities in the labour market. Globally, women represent 42% of the workforce but occupy only 31.7% of senior leadership positions. These disparities are even more pronounced in technical roles and STEM fields, where women hold only 29% of entry-level positions and just 12.2% of executive roles. The lack of full technical contributions from women in scientific fields of animal science, where technological solutions like AI and Agriculture 4.0 are advancing rapidly, limits the ability to find the innovative and sustainable solutions our planet needs.

The gender gap persists prominently in the context of academic and research environments [5,6], ranging from universities to scientific associations. The adoption of gender policies specifically focused on universities and research is relatively recent. Starting in 2022, participation in the new European Framework Programme for Research and Innovation, Horizon Europe, requires public entities, research organizations, and higher education institutions to have a Gender Equality Plan. This requirement was introduced in the “Gender Equality Strategy 2020–2025” by the European Commission [7]. Despite this, concrete advancements in structural changes or the reduction in gender gaps in academia remain a challenge. According to Gallego-Moron and Matus-Lopez [8], university measures to address gender equality vary widely, often shaped by factors like budget constraints and institutional commitment. As a result, these strategies are frequently inadequate and ineffective in achieving meaningful progress. Moreover, even when policies are in place, they appear to have limited results because they mainly address isolated factors rather than taking a multifaceted, integrative approach [9]. For instance, simulations based on Italian data revealed that policies such as compensating for women’s reduced productivity during childrearing years, ensuring greater gender balance in promotion evaluation committees, and implementing gender quotas are not fully effective unless they are implemented together in a comprehensive approach [10]. This highlights the importance of continuing to monitor and critically assess the effectiveness of such policies, particularly in highly specialized fields close to STEM, like animal science, where disparities persist.

This paper seeks to address these challenges by analyzing the current state of gender equality within the field of animal science in Italy, with a particular focus on the academic and research contexts. Specifically, it aims to explore the factors contributing to vertical and horizontal segregation and differences in research performances and identify possible actions to foster greater gender equity.

This preliminary study analyses the current gender scenario within the Italian Association for Science and Animal Production (ASPA), which includes researchers from all Italian universities and research organizations (e.g., National Research Council, etc.) dedicated to various aspects of animal science, including livestock production, aquatic animals, pets, sports animals, and wildlife. The ASPA, with its member base and study commissions, endeavours to advance the field of animal sciences and technologies. Its cultural and scientific role extends further, promoting the exchange of knowledge among Italian scientific communities. Indeed, the ASPA is committed to improving scientific communication within Italian society, emphasizing the importance of addressing gender disparities in academic and research environments.

The authors of this paper are the proponents of the ASPA Working Group on gender equity with the aim of fostering awareness among members, promoting and disseminating gender balance through monitoring the situation within the ASPA, and encouraging the development of a constructive debate aimed at identifying useful actions to overcome persistent vertical and horizontal segregation. To the best of our knowledge, this is the first study to analyze the gender equality scenario within the ASPA.

## 2. Materials and Methods

### 2.1. Collection of ASPA Member Data

The data on the ASPA members were obtained from the association archive between March and April 2023 and include the following information: name, surname, age, gender, type of membership, position, and affiliation organization. The data for 2024 are not yet available.

The academic positions and scientific sectors of each ASPA member were collected by consulting the archive of the Italian Ministry of University at https://cercauniversita.cineca.it/php5/docenti/cerca.php (accessed on 11 April 2023). In Italy, Scientific Disciplinary Sectors (SDSs) denote specific fields or disciplines of focus for scientists and researchers. These sectors serve to organize and classify academic and research activities. Each SDS corresponds to a distinct area of knowledge, allowing for a systematic categorization of research and expertise. Scientists specialize in one SDS based on their research interests and expertise. Within these SDSs, scientific productivity is evaluated by qualifying competitions based on the possession of different threshold values for different SDSs.

In the field of animal sciences, the Scientific Disciplinary Sectors (SDSs) dedicated to animal husbandry encompass the following:

- Animal breeding and genetics, abbreviated as AGR/17;
- Nutrition and feeding, abbreviated as AGR/18;
- Husbandry techniques, abbreviated as AGR/19;
- Short-cycle animals (fish, poultry, rabbit, laboratory animals), abbreviated as AGR/20.

For the academic regular and aggregate members, the Glass Ceiling Index (GCI) was calculated. The GCI is a relative index comparing the proportion of women in academic environments (grades A, B, and C) to the proportion of women in top academic positions (grade A positions, equivalent to full professorships in most countries) in a given year [11]. The GCI can range from 0 to infinity. A GCI of 1 indicates that there is no difference between women’s and men’s promotion prospects. A score of less than 1 means that women are over-represented at grade A level and a GCI score of more than 1 points towards a glass ceiling effect where women are under-represented in grade A positions. In other words, the higher the value of GCI score, the stronger the glass ceiling effect and the more difficult it is for women to move into a higher position.

Key bibliographic parameters (n. of publications, h-index, and citation index with and without self-citations) for each ASPA member were collected from Scopus, one of the largest databases containing abstracts and citations of peer-reviewed research literature (consulted at the end of March 2023). The h-index, also known as the Hirsch index, is a metric used to measure both the productivity and impact of researchers’ scientific output. The h-index provides a single number that reflects both the quantity (number of publications) and impact (citation counts) of a researcher’s work, commonly used as a measure of productivity and influence within academic and research environments.

### 2.2. Data Collection of Authors with Publications on IJAS

The *Italian Journal of Animal Science* (IJAS), the official open access journal of ASPA, invites all members of the association to support the journal by submitting their manuscripts. Starting from February 2023, IJAS articles authored by Italian researchers, both ASPA members and non-members, were extracted from the Web of Science database [12] for four different years (2002, 2008, 2014, and 2021), refining the search by country/region (Italy). This study identified the prestigious positions of first, last, and corresponding authors, and the number of women and men in these roles was counted for each publication. The corresponding author was only counted if different from the first and last author. Gender determination required access to the full name of each author, including their given name in full (not merely initials) and surname. In Italy, gender can be easily inferred from the full name. In the few ambiguous cases involving foreign co-authors, a specific online search was conducted to identify the researcher’s gender.

## 3. Statistical Analysis

The analyses of bibliographic parameters were conducted on a subset of 293 members, excluding honorary members (aged over 70 years), non-academic members, and members who lacked an indication of a Scientific Disciplinary Sector.

Subsequently, these data were analyzed using JMP® Pro 17.0.0 software, utilizing descriptive statistics, ANOVA and ANCOVA models, and non-parametric tests (Welch’s test ANOVA). These analyses aimed at assessing the effect of gender, age, position, and scientific field on bibliographic performances. The Logworth statistics (log transformation of *p*-value) are reported to explain the contribution of each variable to the ANCOVA models used.

The sample size in the different analyses could be smaller in the different analyses reported due to the lack of some information (age, SSDs) for some members or categories analyzed. For example, the PhD students and fellows do not have an indication of SDs.

## 4. Results and Discussion

### 4.1. Gender Distribution of Members in the ASPA

**Figure 1:**
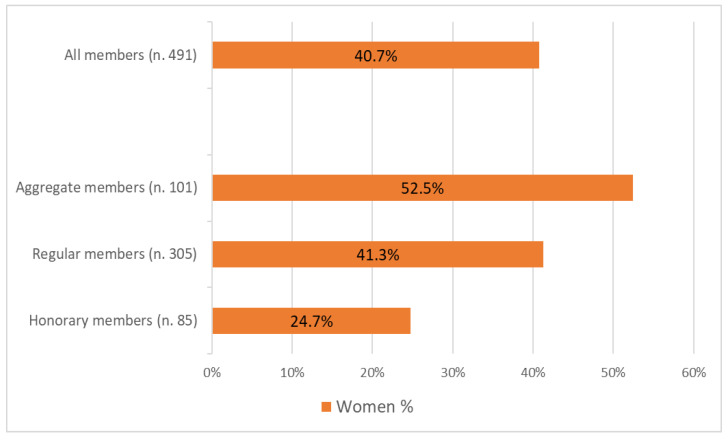
Percentage of women members of the Association for Science and Animal Production in the year 2024.

In 2023, the Association for Science and Animal Production counted a total of 491 members, considering only individuals and excluding organizations. Members were classified into three categories: honorary members (retired members), regular members, and aggregate members. According to ASPA’s statute, aggregate members are young individuals undergoing training in animal science research, including doctoral students, research fellows, and grant holders. Upon completing their training and transitioning to stable positions as researchers, they become regular members. Figure 1 illustrates the percentage of women within the entire association and across the three membership groups. When considering only aggregate and regular members (excluding honorary members), women account for 46.5%. Among aggregate members, the proportion of women is 52.5%, markedly exceeding the overall average.

According to statistics from the European Union (EU), the proportion of women among doctoral graduates has shown a slight increase in the last decade, reaching 48.1% in 2018 (EU-27) [11]. This outcome suggests that, without differentiation by the field of study, gender parity between female and male doctoral graduates has almost been achieved at the European level, indicating gradual progress towards gender equality over time. At the Italian level, the proportion of female doctoral graduates is slightly higher than at the European level, although the evolution between 2010 and 2018 recorded a slight reduction in the percentage of young women obtaining a doctoral degree, from 53.2% to 50.5% [11]. In particular, when examining the specific fields of “Agriculture, forestry, fisheries and veterinary”, which are closely related to but not completely overlapping the field of animal sciences, the percentage of women gaining a doctorate in 2018 was 56.8% at the European level (EU-27) and 56.3% at the Italian level. These figures are slightly higher than the 52.5% percentage of aggregate members of the ASPA, which also includes research fellows.

The ASPA was established just over 50 years ago (1973), with only 3 out of its 28 founding members (10.7%) being women. Over time, the percentage of women in the association has steadily increased, rising from 20.6% in 1994 to 40.9% in 2023, reflecting a nearly threefold growth over 30 years.

The association primarily includes scientists from all Italian universities and other research organizations, along with a small number of technicians and professionals. Its members are distributed across eighteen regions in Italy. Membership is exclusively male in two regions, while thirteen regions exhibit a male majority, one achieves gender parity, and two have a female majority.

Academic members constitute the vast majority, representing 88.2% of the association when excluding honorary members. In contrast, among non-academic, non-honorary members, gender parity (50:50) is observed, demonstrating a more balanced distribution compared to their academic counterparts.

The academic members of the association represent almost the entire cohort of Italian academics within the field of animal sciences and technologies, which totals 409 scientists, 46.7% of whom are women.

**Figure 2:**
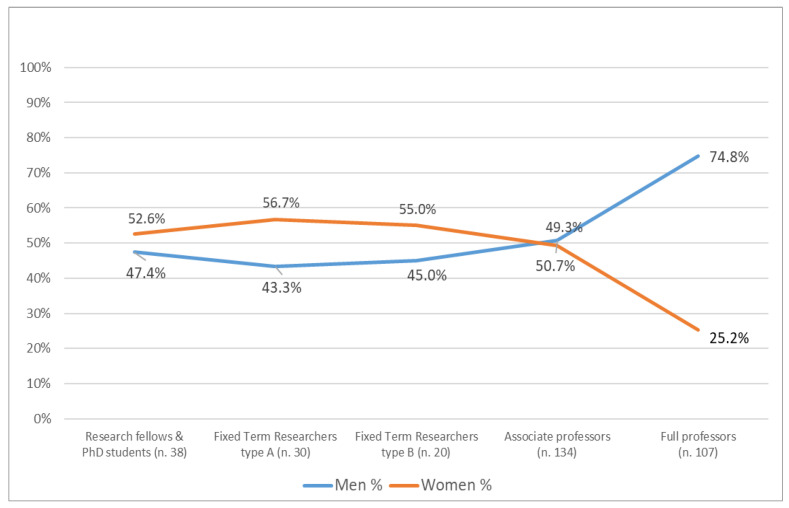
Members of the Association for Science and Animal Production in the year 2023 by position and gender. Only academic regular and aggregate members, excluding tenured researchers, were reported.

When classifying academic members (aggregate and regular members) by position and gender, a notable gender disparity emerges in the full professor position (Figure 2). The figure does not include the category of tenured university researchers as this category is being phased out and is no longer renewed. Notably, women represent 57% of the tenured researcher category.

The diagram highlights the typical “scissor-like” trend that characterizes the male-to-female ratio at different career levels: the female component consistently dominates in lower-level positions but becomes a minority in higher-level positions, as confirmed also by the glass ceiling index, which is equal to 1.7. The balance point corresponds to the position of associate professor. This trend, with some differences, is also confirmed at the national level concerning academic staff in 2020 [13]. A similar trend among academic staff is also reported at the European level [14]. According to this report, while the overall percentage of women in academic roles is generally close to parity, the representation of women significantly decreases in higher positions. In 2018 (EU-27), the proportion of women declined from nearly half (46.6%) in grade C positions (PhD holders) to 40.3% in grade B (more senior) positions. There was a further decline to approximately one-quarter of women (26.2%) in the grade A positions, which represents the highest grade at which research is typically conducted within the institutional or corporate system. Moreover, it should be emphasized that from 2015 to 2018, the improvement at the European level appears minimal, indicating a prolonged path toward gender parity. In Italy in 2018, only 23.7% of grade A positions in academic environments were held by women.

The gap is particularly noticeable in Scientific Disciplinary Sectors compared to social and humanities disciplines: the proportion of women in category A was 17.9% and 28.5% in Engineering and Technology and Agricultural Sciences, respectively, while, in social and humanities sciences, it exceeded 30% in 2018. In Italy, the situation appears even more imbalanced, with only 13.8% and 19.5% of grade A positions being held by women in the fields of Engineering and Technology and Agricultural Sciences, respectively [11].

The “scissor effect” refers to a prevalent trend of vertical segregation observed across various work domains. However, as highlighted by Farina et al. (2023) and other authors, gender disparities are particularly pronounced in the academic and research spheres, with progress toward overcoming these inequalities advancing at an exceptionally slow pace [14]. The representation of women declines as if there were a “leaky pipeline” during advancement from junior to senior academic positions, with high attrition rates observed [15]. The under-representation of women in higher career positions can be attributed to both the “leaky pipeline” and “glass ceiling” phenomena. The former refers to the effect of women leaving the career pipeline at various stages while the latter pertains to structural barriers such as discrimination and gender bias that hinder women’s access to top decision-making and managerial roles [11].

The causes of vertical segregation in academic careers are multifaceted, complex, and interconnected. On one hand, there is a well-documented, lower scientific productivity among women compared to men based on commonly used indicators for assessing scientific output: the number of publications, citations, and citation indexes [16]. The lower scientific productivity of women could be partially attributed to the greater family responsibilities of women, not only childcare but also caring for elderly and disabled family members. These family commitments compete with research activities but also limit opportunities for travelling and the establishment of national and international networks, both crucial factors for accessing funding and publishing scientific work. It is not coincidental that the international mobility of European female doctoral candidates is higher than that of their male counterparts, while among senior researchers, the ratio is reversed, with men exhibiting greater international mobility [11]. The lower mobility of female senior researchers translates into fewer international contacts and opportunities to secure international grants with international teams, leading to diminished chances of publishing and lower visibility in the academic world.

Moreover, within the academic context, women tend to exhibit a stronger inclination than men towards educational activities (teaching and mentoring) and administrative tasks, thus diverting time away from research [17,18]. In addition, several authors have highlighted that female researchers may be more hesitant to participate in competitions due to a lack of self-confidence [19].

According to Filandri and Pasqua (2021), who analyzed career advancement using data from the entire population of researchers and professors in the Italian university system, a significant gender gap in career progression emerges [18]. The analysis revealed that, in this case, the gap cannot be attributed to lower scientific productivity or women’s reluctance to seek promotions. Instead, it exposed structural gender biases. These biases, partly conscious and partly unconscious, are likely to contribute to undervaluing the performances and scientific competencies of women. For instance, the study by Jappelli et al. (2017) suggests that research papers authored by women receive less favourable evaluations [16]. Bendels et al. (2018), analyzing 295,557 research articles from 54 journals listed in the Nature index, highlighted that women are authors of fewer articles than men (two articles fewer per year), and articles with female key authors are cited less frequently [20]. The gender difference in citation rates increases as the number of authors contributing to an article increases. The authors concluded that the prognosis for the coming decades predicts a really poor harmonization of disparity between the two genders. Furthermore, additional barriers to career progression include working cultures that lack gender sensitivity and fail to accommodate family commitments, incidents of sexual harassment, bullying, gender-based violence, and differences in individual choices and behaviour based on gender [13].

As a result, lower proportions of women, relative to men, attain full professorship positions, which are considered as a pre-requisite for top-level decision-making roles such as faculty heads or university rectors. These positions are generally associated with “desirable” attributes, including higher salaries, prestige, social security, and adequate pension benefits. In the academic context, the over-representation of men among heads of universities is an example of such vertical segregation. A South Korean study found that gender quotas improved female faculty representation at all tenure levels but not in senior leadership roles like the dean or president. This suggests that entry-level quotas may not suffice to address gender inequality at higher academic levels. Evidence of their effectiveness across disciplines indicates limited and slow progress in fields where women are severely under-represented [21].

**Figure 3:**
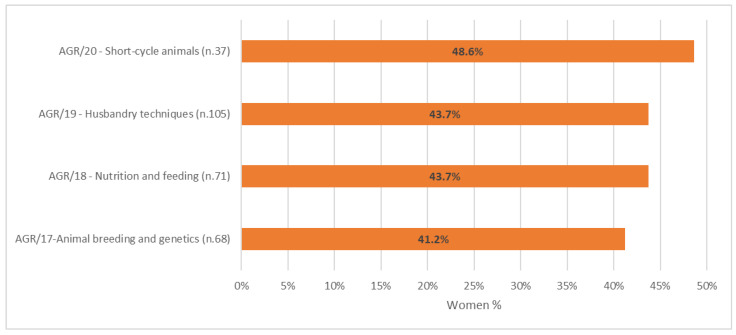
Percentage of women among the academic members of the Association for Science and Animal Production in the year 2023 by the main scientific disciplinary sectors. Only associate and regular members with SDSs were reported (*n* = 281).

In addition to vertical segregation, horizontal segregation was investigated by examining the gender distribution across Scientific Disciplinary Sectors among the association’s members. The analysis revealed a clear predominance of men in disciplines related to Animal breeding and genetics (AGR/17; see Figure 3), which are more closely associated with STEM fields. Values indicating closer gender balance were observed in disciplines related to Short-cycle animals, such as fish, poultry, laboratory and fur animals, birds, and rabbits (AGR/20).

Horizontal segregation refers to the concentration of women and men in different sectors (sectoral segregation) and occupations (occupational segregation) [22]. It can occur within education with over- or under-representation of one gender in specific subjects and employment with over- or under-representation of one gender in specific professions, industries, etc. The issue of horizontal segregation may in turn lead to increased vertical segregation. For instance, undervaluing competencies associated with “women’s work” may limit women’s prospects for career advancement [11].

Out of the 281 academic regular members, 44% are women. Within the SDSs, women represent 41.2% in Animal breeding and genetics and 48.6% in Short-cycle animals.

**Figure 4:**
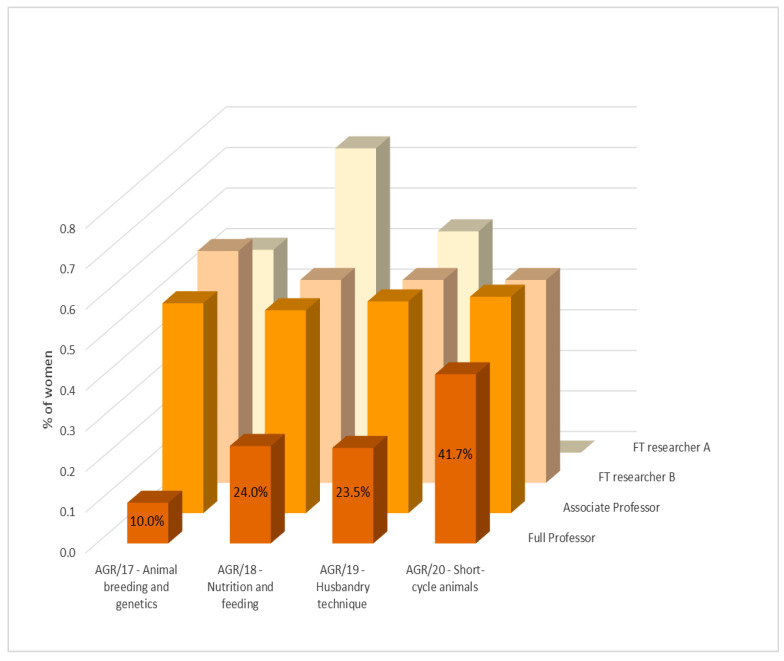
Percentage of women among the academic members of the Association for Science and Animal Production in the year 2023 by the scientific disciplinary sectors and position. Only regular and aggregate members, excluding tenured researchers (n. 281). FT = fixed-term.

Although in the field of Animal breeding and genetics (AGR 17) a smaller percentage of women is observed, this value is not significantly different from the overall average of 44% of women across all SDSs. However, it can be noticed that in three out of the four SDSs considered (AGR17, AGR18, AGR19), there is a strong vertical segregation, with the number of female full professors significantly lower compared to men. Only in the AGR20 sector, gender differences across academic positions are minimal and not statistically significant. In the field of Animal breeding and genetics (AGR/17), the strongest vertical segregation is observed, with a very low percentage of women in full professor positions (10%) compared to other SDSs (Figure 4), probably linked to a stronger gender prejudice that considers statistics and calculation as a prerogative of men, as in other STEM disciplines. This is particularly concerning given the growing reliance of animal science and the wider agricultural sector on precision livestock farming and artificial intelligence tools, making these fields increasingly interconnected with STEM disciplines. A lack of female representation may limit diverse perspectives in a sector that is advancing rapidly through technological and scientific innovations. This perspective is invaluable as we aim to develop innovative solutions that not only increase productivity but also support broader goals of ecological and social sustainability [23]. In-depth analyses of the role of women in promoting environmental and social sustainability have been published recently in various fields [24,25].

The percentages of women reported in Figure 4 reflect the proportion of women present in the four SDSs at the national level in the Italian university system. Overall, women constitute 45.9% of the total, and the percentage of women among full professors is 12.5% in AGR/17, 30.8% in AGR/18, 26.3% in AGR/19, and 58.3% in AGR/20, based on data from the official website of the Italian university system [26,27]. It is worth noting that the EU defines gender parity as a 50:50 ratio and gender equality as a proportion between 40 and 60%. In the case of full professors, there is an evident inequality as confirmed by the glass ceiling index which exceeds 1 in three out of the four SDSs.

Vertical segregation in the SDSs of animal science in Italy could be calculated as the proportion of women and men who are full professors over the total number of women and men in academic environments (from full professors to tenure track researchers and fixed-term type B and A researchers). This results in a significant difference (*p*-value = 0.0014) with percentages of 15.7% and 34.3% for women and men, respectively. The opportunity for women to become full professors, calculated as relative risk, was 0.44 in 2023, whereas, in 2013, it was 0.27 (when the percentages of women and men full professors were 9.6% and 35.2%).

Vertical segregation could also be seen from the glass ceiling indexes, equal to 2.2 for AGR/17, 1.8 for AGR/18, 1.6 for AGR/19, and 0.9 for AGR/20. These indexes show that in three out of the four SDSs with a value higher than 1, a strong vertical segregation is currently present. The GCIs for the above sectors in 2013 were 1.7, 2.2, 5.3, and 1, respectively. Although in some SDSs, the GCI has improved over the last 10 years; by calculating the GCI for the four SSDs together in the same 10 years, it moves from 1.7 to 1.6.

When comparing the values of 2023 with those of 2013, there has been an increment of approximately 0.6% per year in the number of women full professors and a decrease of 0.1 in the GCI. It can be estimated that it will take approximately 60 years, given the same trends, to have equal opportunities for women and men to access top positions in the four SDSs in animal science.

The probability for a woman to become a full professor is significantly lower (*p*-value < 0.05) in three out of the four SDSs evaluated. Women (and men) in AGR17, 18, and 19 show values of 7.7% (40.4%), 17.4% (38.3%), and 15.9% (34.1%), respectively. Only in the AGR/20 sector is there a higher but not statistically different value for women (vs. men), equal to 23.3% (17.2%). Overall, women have a probability of becoming full professors equal to 0.44 while men have 2.3 times that probability. These values underline a persistent inequality of opportunity for women to reach the position of full professor, contributing to a gender gap in salaries and retirement pensions that accumulate disadvantages throughout the entire career of a woman scientist. It would also be interesting to evaluate these data with respect to academic age, defined as the age at which individuals achieve different career milestones; however, the necessary data are not available.

While animal sciences, as a whole, may not fall entirely within the STEM category (Science, Technology, Engineering, and Mathematics), certain disciplinary areas within it encompass STEM skills. Research in the field of Animal breeding and genetics now demands proficiency in mathematics, statistics, and computer science. Despite improvements in recent years, women working in STEM fields are still in the minority. According to the 2023 report on gender equality in the EU [26], the proportion of women graduating in STEM disciplines is persistently lower than that of men and is relatively stable: there are two men graduating in tertiary education in STEM disciplines for every woman. The ratio is slightly better at the master’s or equivalent level and at the doctoral level (respectively, 1.5 and 1.8 men for every woman in 2020) [27].

As previously highlighted, when discussing doctoral graduates, at both European and Italian levels, women graduates were over-represented in the field of Education and under-represented in the broad fields of ICT and Engineering, Manufacturing, and Construction [11]. For instance, in Italy, out of 100 female students who enrol in university, only 21 choose STEM degree courses. Moreover, among all those enrolled in STEM faculties, female students represented only 37% [28]. This gender gap is present not only in Italy but also in Europe and around the world. As teaching and learning cultures can be influenced by gender stereotypes and unconscious biases, this may constitute a barrier to women’s progression in STEM fields [29]. Some of the contributing factors to this situation include gender stereotypes, lack of female role models in STEM disciplines, and differences in education and perception of these disciplines [30].

In the EU, men dominate 85% of all top-level positions in STEM fields [28]. These data suggest that the extent of vertical segregation in career paths for women in academic environments is more pronounced in the field of STEM. Particularly in STEM, women face barriers including biases from hiring committees, lack of mentoring, social marginalization, inhospitable group cultures, lower salaries, fewer resources, less respect, lower likelihood of promotion, and even overt opposition to hiring female faculty members [31].

### 4.2. ASPA Governance and Committees

Regarding aspects related to the governance of the association, since 1973, the year of its foundation, ASPA presidents have always been men, and the board of directors has had an almost exclusively male composition. Only since 2013 has the board, consisting of five members, including the president, seen the presence of one or two female members. This phenomenon is largely associated with the different proportion of women reaching the position of full professor because, as previously highlighted, certain apex positions and roles are almost exclusively held by full professors.

The ASPA also has permanent or temporary internal study committees that look into specific topics within the field of animal production and promote activities such as seminars, conferences, and scientific and informative publications. These committees arise from a proposal by a member, which is first examined by the Board of Directors and then circulated among the other members. Membership in these committees is voluntary but regulated to avoid overly large groups. It is possible to be part of more than one committee. In 2023, ASPA hosted 11 study committees with a total of 168 members, averaging 15.3 members per committee, from a minimum of 8 to a maximum of 26. Women constitute 40.5% of the committee members and 41.7% of the committee coordinators. Since membership is voluntary, it was expected that the percentage of women in the committees would be similar to the overall percentage of women in the association. However, it is noteworthy that even within the committees, evidence of vertical segregation is apparent, with a higher female presence in the study committee on “Companion animal breeding” (75%) and a lower presence in committees dedicated to more technical topics such as “Precision Livestock Farming” and “Statistical Methodology and Experimental Design” (18.8 and 23.1% resp.).

### 4.3. Bibliometrics of the ASPA Members

The scientific performances of the ASPA academic members belonging to the three main categories (fixed-term researchers type A and B, associate professor, and full professor) were analyzed by examining key bibliometric parameters (no. of publications, h-index, no. of citations, and no. of self-citations). These data were collected by Scopus in March 2023.

When considering only gender as a source of variation, statistically significant differences between women and men are observed for the four variables considered and age (Table 1). Men outperform women in all the variables considered, with a statistically significant average (and median) difference in favour of men equal to 17.3 (9.5) documents, 2.5 (2.0) points of h-index, 391.5 (189.5) citations, and 66.3 (13) self-citations. Moreover, the average age of male authors is 3.5 years higher than that of females. This model explains only 2.3 to 3.3 percent of the observed variability. The differences remain significant even when using Welch’s test for the variables that show unequal variances (publication numbers, citations, and self-citations).

**Figure 5:**
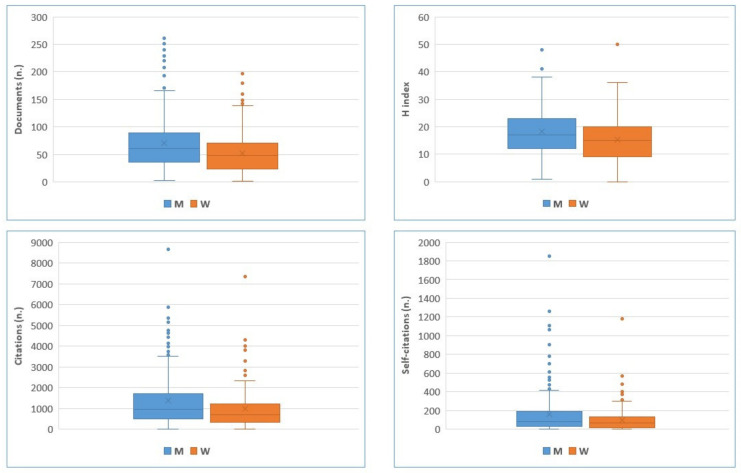
Distribution of bibliometrics by gender for ASPA academic members. The whiskers of the box plot extend from the box to the minimum and maximum data points that are not considered soft outliers (outside 1.5 times the Interquartile indicated by dot).

This effect is clearly shown in Figure 5, where a higher number of male outliers in terms of the number of documents can be observed. For this metric, out of the 24 top researchers in the first 10% of the distribution, 33% are women. All of the top male researchers hold full professor positions, whereas among women, only 75% are full professors. This observation aligns with findings from previous studies [32], indicating that gender disparities in academic environments can largely be attributed to the extremes of the distribution, notably the disproportionate representation of men among the highest-achieving scientists.

Owing to the low variability explained by the previous model, which considers gender as the sole source of variation, a three-way ANCOVA model was applied, including gender, academic position, and scientific sector as fixed factors with age as a covariate.

It is worth noting that a logarithmic transformation for the variables citations and self-citations was also explored to address the funnel pattern observed in the residuals. However, this transformation did not alter the significance of the factors. Firstly, a saturated model incorporating two- and three-way interactions was applied, but none of these interactions proved statistically significant and were thus removed from the model.

With this model, gender consistently showed no significance for the four bibliometric variables considered (Table 2). On the contrary, academic positions, including fixed-term researchers type A and B, associate and full professors, consistently yielded high significance, emerging as the most important source of variation, as demonstrated by the highest Logworth value. In addition, the SDSs were significant, although the Logworth was lower, except for the h-index. The covariate age also proved significant for all the metrics considered, with Logworths lower than those for the SDSs. The r2 of the model increased for all four variables, with the highest value (0.36) for the h-index and the lowest (0.21) for the self-citations.

**Figure 6:**
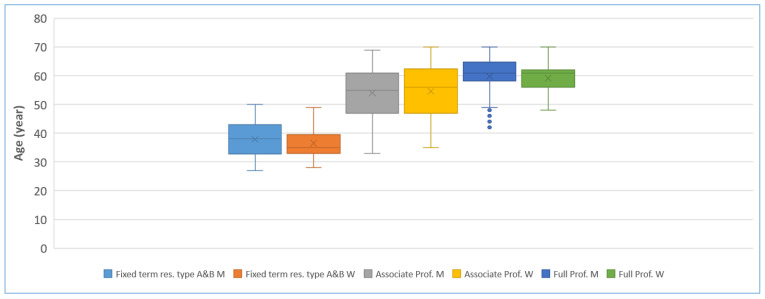
Age by position and gender. The whiskers of the box plot extend from the box to the minimum and maximum data points that are not considered soft outliers (outside 1.5 times the Interquartile indicated by dot).

The results concerning positions and age align with expectations: as careers progress, the parameters under consideration tend to increase, as illustrated in Figure 6. Notably, among male full professors, there are more outliers in the younger age bracket, hinting that men often advance more swiftly in their careers and attain higher positions earlier compared to women. Several longitudinal studies have linked the gender gap in bibliometric performance to women’s shorter career durations [33], a higher representation of women in teaching-intensive and part-time positions [34], and fewer women in the top positions where both genders achieve the highest performance [35].

Analyzing a model that incorporates academic age at various career stages by gender would be beneficial, but regrettably, such data are unavailable.

The position emerged as the most crucial factor. Indeed, the exclusion of the full professor category, dominated by older men at 75%, eliminates statistical gender differences. On the other hand, when considering age alone, gender becomes statistically significant, although with a low explained variability when using gender alone in the simplest model.

**Figure 7:**
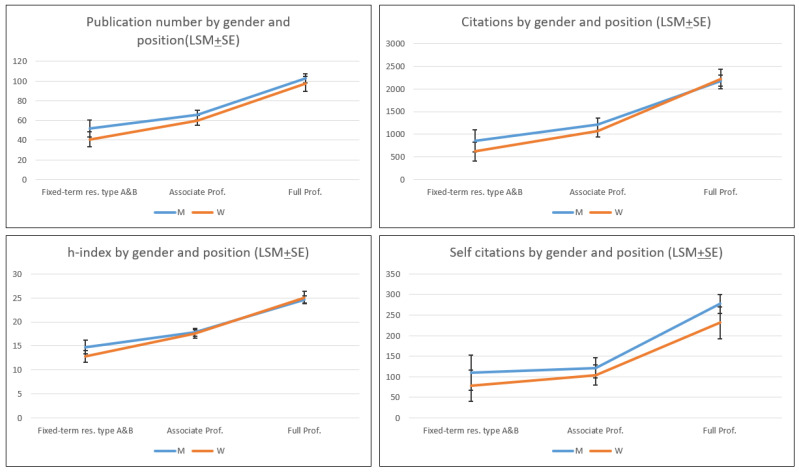
Least Square Means (LSM ± SE) of the bibliometric parameters by gender and position.

In Figure 7, where the Least Square Mean (LSM ± SE) by positions and gender are reported, it is evident that gender is not statistically significant. 

Although differences by gender are not statistically significant (Figure 7), the main descriptive statistics of the distribution by gender and position (Table 1 and Supplementary Table S1) show differences starting from early career stages. These disparities persist across the higher positions, although diminished for some metrics. This pattern highlights both the “sticky floor” and “glass ceiling” theories, especially evident in outliers among young male full professors but absent among young female full professors.

To summarize, considering both academic positions and age, statistical parity emerges between men and women in terms of performance. However, academic positions appear as the primary determinant of bibliometric performance. Specifically, the early attainment of full professor positions enhances opportunities for securing research funding, fostering collaborations, and building professional networks.

As Abramo et al. (2021) note, an intriguing question arises: could the employment of bibliometric indicators contribute to a more equitable assessment of female researchers? [32] Various strategies have been suggested, with the most radical proposal put forward by Abramo et al. (2016) [35], involving the establishment of performance rankings that differentiate between genders. This proposal stems from the observation, corroborated by our own data, that “when comparing the research performance of individuals within the same gender category, distinct rank positions emerge compared to those rankings that disregard gender distinctions”.

This concept parallels the practice in sports, where gender-specific rankings are common to ensure fair competition and recognition of individual achievements within distinct categories.

### 4.4. Gender Distribution of Authors on IJAS

The number of papers published on the IJAS by Italian scientists increased over the years (from 27 to 75 papers in the last two decades). In 2002, only 30% of authors were women and the percentage of prestigious positions covered by female scientists was 22%, the lowest value obtained in the four years included in the survey.

The gap between the percentage of female authors and the prestigious positions held by women widened even more in 2008 (−13%) when about one-third of women covered prestigious positions even though the percentage of female authors was nearly 50%. In 2014, the total number of papers published on IJAS increased significantly in comparison to 2008 but the gender distribution remains unchanged. A trend towards achieving a gender balance was observed only in 2021, with a sharp increase in prestigious positions covered by women (41%) despite the lower percentage of female authors (36%) compared to the two previous surveys.

Abramo et al. (2009) studied the research productivity of the Italian academic system between 2001 and 2003 using the Italian Observatory of Public Research (ORP) [36]. The percentage of Italian female scientists in the disciplinary area of Agriculture and Veterinary Sciences was 27%, and this value is close to the gender distribution of authors in IJAS in 2002 (30%). The authors discovered that in the same period, women were more active (more than one paper per year) than men (52.7% compared to 47.5%). In the report by the European Commission (2018), the women-to-men ratio of authorship in the field of Agricultural Sciences (including animal science) increased from 0.9 to 1.0 in Italy between the five-year periods 2008–2012 and 2013–2017 [30]. These ratios are similar to the value found in this study in 2008 (0.92) but lower than that of 2014 (0.72), which is closer to the EU-28 ratios (from 0.7 to 0.8 in two periods). According to Ross et al. (2022), women in research teams are significantly less likely than men to be credited with authorship [37]. An investigation into the gender balance in STEM subject areas in Portugal showed that, among all researchers in any author position, women wrote fewer publications than men and this gap was largest in 2003 or earlier, although it decreased over the years, reaching parity in the five-year period 2014–2018 [38]. In addition, Bendels et al. (2017), analyzing almost 300.000 articles from 54 journals listed in the Nature index from 2008 to 2016, found that women comprise, on average, 47.6% of all authorship in Italy [20]. This value is very similar to that obtained in this study considering the two central years (2008 and 2014).

The percentage of female first authors increased sharply from 2002 to 2008 (15 to 41%) and this increase continued linearly in 2014 and 2021 when the 50% threshold was reached. Therefore, the growing role of women in the authorship of scientific articles has been confirmed over the years. Conversely, the percentage of women as last authors remained stable or decreased over time (from 30 to 25%). Typically, the last position is held by the author responsible for acquiring funds and other resources necessary to carry out the experiments. Therefore, these data may reflect some difficulties of women in accessing public and private funding and performing the role of principal investigator.

Johnson et al. (2021) observed that the percentage of reviewed research abstracts with a female last author, during the period 2006–2018 at the Pediatric Orthopedic Society of North America annual meeting, was very low (17.8% of the total) but, in addition, abstracts with women as last authors were significantly less likely to reach publication compared to those with men (59.6 vs. 67.9%; *p* < 0.05) [39]. In the paper by Bendels et al. (2017), women are, on average, 33.1% of the first authors and 18.1% of the last authors at the global level [20]. The highest average annual growth rate from 2008 to 2016 was found for the last authorship (1.4%) followed by the first authorship (0.7%). This positive trend is confirmed in this study only for the female first authors while, for the female last authors, the percentage is stable (on average 25% in the last three years considered).

Nevertheless, the percentage of women in the role of corresponding author (considered only if this position is different from the first or last author) increased threefold (from 15 to 45%) over the years. This trend is not linked to a corresponding increase over the years in the total number of women involved in the publication of articles (+6%) and could be justified by the fact that women are traditionally believed to have a greater aptitude for managing, rearranging, and proofreading manuscripts, following the stereotype that women are more organized and more persevering than men. This gender stereotype is derived from the unequal distribution of men and women in social roles both in the household and the workplace [40].

In a recent publication on gender disparities in high-quality research revealed by the Nature index, the writers, who did not have access to the researchers’ academic positions, reported lower productivity for female authors [20]. In the same publication, the writers also reported that articles with female key authors (first and last) are cited less frequently than articles with male key authors.

This difference in citation rates increases as the number of authors contributing to an article increases, probably due to the strong competition for key positions among female authors. Furthermore, they observed distinct differences at the level of journal, journal category, continent, and country.

## 5. Conclusions

This study highlighted that within the association for animal sciences and the broader field of animal sciences, there is still a noticeable gender gap that has reduced over the years but at a very slow pace. In particular, there is a vertical segregation with a minority of women occupying full professor positions and a horizontal segregation with a low percentage of women involved in technical disciplines closer to STEM. At the bibliometric level, differences in bibliometric indicators between women and men are observed, favouring the latter, although these differences are not significant when taking into account academic position and scientific sector as fixed factors, with age as a covariate. Considering the authorship of scientific papers, women are under-represented in prestigious positions such as the first, last, and corresponding author.

This study provides evidence of barriers hindering women’s advancement in science despite recent policy efforts for equality. However, some limitations must be acknowledged. It focuses on a single disciplinary field, examines only the Italian context, and does not include variables such as academic age due to data unavailability. Despite these constraints, the findings offer valuable insights for understanding gender disparities in academia.

The question of why so few women reach top positions and why progress is slow remains. The roots of this imbalance are deeply entrenched in local and historical contexts, perpetuating systemic inequalities. Effective policy efforts to increase women’s participation in science must address complex social, cultural, economic, and political factors. Each country must identify and tackle the mechanisms perpetuating past inequalities, as neglecting the intellectual contributions of half the population cannot be justified.

Overcoming the gender gap in academic and research environments requires action not only within universities, research centres, and associations but also in broader societal realms. Cultural evolution, education of new generations against stereotypes, family support, and welfare policies are essential. However, the academic and scientific community is also called upon to play its part in the process toward gender equality, a process that is currently advancing too slowly. Efforts should prioritize equity in power roles within institutions, visibility in scientific events, and authorship in scientific publications. Research groups should strive to achieve gender balance across all disciplines, and balanced teams should be incentivized. Departments and universities should be encouraged to adopt resource distribution criteria that consider gender balance in leadership and decision-making roles. Furthermore, selection panels for research projects and editorial boards should ensure balanced gender representation and receive training to mitigate biases that contribute to the under-representation of women. As highlighted by several authors, the simultaneous implementation of all policies is essential; otherwise, progress may be significantly delayed. Scientific associations, such as universities, should adopt regular gender balance monitoring, using indicators to measure and drive progress. Establishing an annual evaluation system tailored to gender in academic environments could aid in addressing the issue.

## Figures and Tables

**Table 1:** Main descriptive statistics for biometric parameters and age by gender.

	Gender	Women	Men	(W-M)	Statistical Parameters	
Publication	Sample size (n)	121	172		r2	0.033
	Mean	63.9	81.2	−17.3	*p*-value	0.0006
	Median	57	66.5	−9.5	Welch’s *p*-value	0.0003
	S.D.	34.6	50.1			
h-index	Sample size (n)	121	172		r2	0.026
	Mean	18.1	20.6	−2.5	*p*-value	0.0039
	Median	17	19	−2.0	Welch’s *p*-value	
	S.D.	7.1	8.2			
Citation n.	Sample size (n)	121	172		r2	0.025
	Mean	1224.3	1615.9	−391.5	*p*-value	0.005
	Median	1003	1192.5	−189.5	Welch’s *p*-value	0.0033
	S.D.	1024.6	1376.4			
Self-citations n.	Sample size (n)	121	172		r2	0.023
	Mean	126.6	192.8	−66.3	*p*-value	0.0068
	Median	98	111	−13.0	Welch’s *p*-value	0.0033
	S.D.	140.2	252.7			
Age (years)	Sample size (n)	121	172		r2	0.025
	Mean	51.6	55.2	−3.5	*p*-value	0.0038
	Median	53	58	−5.0	Welch’s *p*-value	
	S.D.	11.3	10.6			

r2: coefficient of determination of the ANOVA model. Welch’s *p*-value is reported when the assumption of ANOVA is not satisfied.

**Table 2:** Effects and r2 of the three-way ANCOVA model used.

Y Variables	Effects				
Obs. n. 280	Positions	Age (Year)	S.S.D.	Gender	r^2^
Document n.					0.32
Logworth	15.13	2.72	2.64	1.01	
*p*-value					
h-index					0.36
Logworth	16.06	4.20	1.28	0.48	
*p*-value	<0.0001	0.0001	0.0530	0.3324	
Citations n.					0.29
Logworth	11.401	3.991	1.022	0.665	
*p*-value	<0.0001	0.0001	0.09511	0.21642	
Self-citations n.					0.21
Logworth	9.32	3.12	1.80	0.80	
*p*-value	<0.0001	0.00076	0.01603	0.15866	

## Data Availability

Data is contained within the article.

## Supplementary Materials

The following supporting information can be downloaded at https://www.mdpi.com/article/10.3390/ani15030390/s1, Table S1: Description of bibliometrics distributions by gender and positions.
