# Supplementary figures and images for: Assessing Gender Equality in Italian Animal Science: A Case Study on Academic Careers and Research Outcomes
Source: Animals (Basel). 2025 Jan 30;15(3):390. doi: 10.3390/ani15030390 (PMC11816178; doi:10.3390/ani15030390)

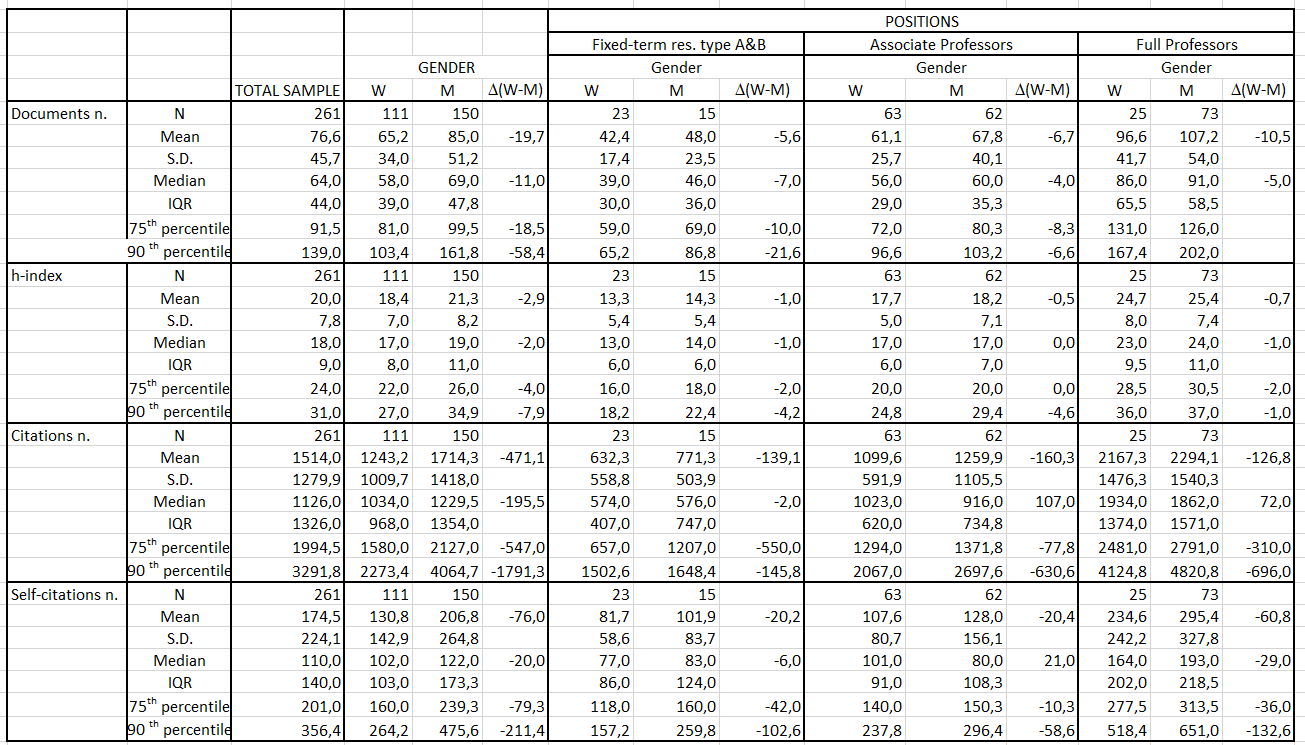

Supplement: Supplementary file 1 [file animals-15-00390-s001.zip › animals-3379756-supplementary.png]
